# One-pot rota-crystallized hollownest-structured Ti–zeolite: a calcination-free and recyclable catalytic material†
†Electronic supplementary information (ESI) available. See DOI: 10.1039/c6sc01735e


**DOI:** 10.1039/c6sc01735e

**Published:** 2016-05-04

**Authors:** Dan Zhou, Tianjun Zhang, Qinghua Xia, Yarong Zhao, Kexin Lv, Xinhuan Lu, Renfeng Nie

**Affiliations:** a Hubei Collaborative Innovation Center for Advanced Organic Chemical Materials , Ministry-of-Education Key Laboratory for the Synthesis and Application of Organic Functional Molecules , Hubei University , Wuhan 430062 , P. R. China . Email: xiaqh518@aliyun.com

## Abstract

A hydrothermal rota-crystallization method is developed for the one-step synthesis of a hollownest-structured zeolite precursor with the shell composed of autogenously-intergrown MWW nanosheet crystals containing a large number of stacking-pores without using any porogen or hard scaffold. This material possesses a large external surface area. By simple acid washing, the resultant Ti-containing catalyst can be directly used in the epoxidation of alkenes with hydrogen peroxide. The excellent catalytic activity over the Ti-HSZ catalyst is assumed to be due to the exposure of more Ti active sites over the MWW nanosheet crystals in the shell of the catalyst. More importantly, this Ti-HSZ catalyst washed with H_2_O_2_/ethanol solution has been reused 6 times without an appreciable decrease in both the conversion of the allyl chloride and the selectivity of the epichlorohydrin, which is ascribed to the structural stability of the hollownest-structured zeolite.

## Abstract 2

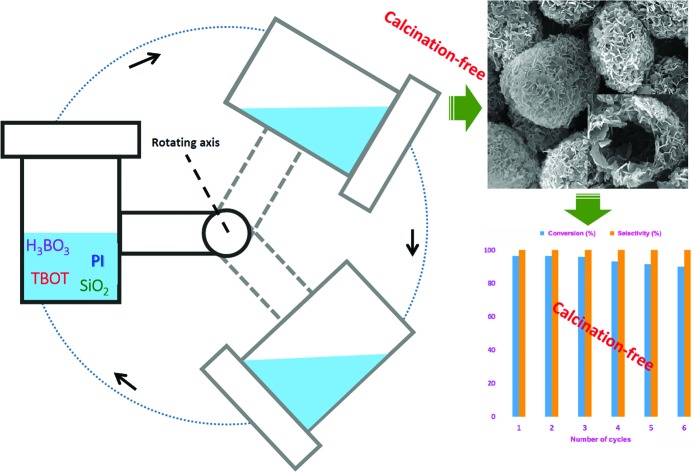
A hydrothermal rota-crystallization method is developed for the one-step synthesis of a hollownest-structured zeolite precursor with the shell composed of autogenously-intergrown MWW nanosheet crystals containing a large number of stacking-pores without any porogen or hard scaffold.

## 1. Introduction

Zeolites, a class of microporous crystalline materials with a pore diameter of less than 2 nm, are the most important type of solid catalysts and have found many applications in the chemical industry.^1^–^5^

The catalytic epoxidation of alkenes is an important reaction in the manufacture of fine chemicals and pharmaceuticals, since epoxides are key building blocks in organic synthesis.^6^,^7^ As one of the most important members of the zeolite family, Ti-containing zeolites have scientific and technological interest due to the excellent properties in the environmentally-benign selective epoxidation of alkenes to epoxides.^8^–^13^ Among the various titanosilicate zeolites, Ti-MWW zeolite has displayed superior catalytic performance in the selective epoxidation of linear or other small alkenes,^12^–^16^ which was first reported by Wu *et al.* However, for conventional direct synthesis and post structural rearrangement methods, a long crystallization time of 7 days and a relatively complex synthesis process are unavoidable.^12^–^16^

**Scheme 1:**
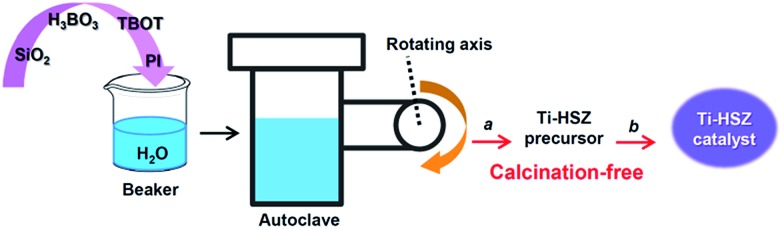
Construction of the Ti-HSZ catalyst by a one-step hydrothermal rota-crystallization method ((a) filtration, washing with deionized water, drying; (b) washing with HNO_3_ and deionized water, filtration, drying).

Herein, through a one-step hydrothermal rota-crystallization method (Scheme 1), we construct a micro–meso–macroporous hierarchical Ti-containing hollownest-structured zeolite (Ti-HSZ) precursor in only 3.5 days without using any porogen or hard scaffold. This method is different from the synthesis approaches of hierarchical zeolite materials reported in the literature.^17^–^21^ Interestingly, this synthesized zeolite contains a microcavity with a large external surface area, and its shell, composed of randomly intergrown MWW nanosheet crystals, shows a hierarchical pore distribution. Importantly, through simple acid-washing, the majority of the piperidine molecules can be removed from the precursor, and the resultant Ti-HSZ catalyst shows an excellent catalytic activity for the epoxidation of alkenes. The catalyst can be reused 6 times without obvious deactivation with regeneration by merely washing rather than by calcination at high temperatures, suggesting a highly recyclable stability of the prepared Ti-HSZ catalyst.

## 2. Experimental section

### 2.1 Raw materials

The chemicals used included fumed silica (aerosil-200 SiO_2_, 99.9%, Degussa), *tetra*-butyl orthotitanate (TBOT, 98%, Feida), boric acid (H_3_BO_3_, 99.5%, Sinopharm), piperidine (PI, 99%, Sinopharm), deionized water, nitric acid (HNO_3_, 66%, Shuangrun), allyl chloride (98%, Aladdin), hydrogen peroxide (H_2_O_2_, 30%, Sinopharm), acetone (99.5%, Sinopharm), and ethanol (99.7%, Sinopharm).

### 2.2 Synthesis of the Ti-HSZ catalyst

The Ti-HSZ sample was synthesized using fumed silica (aerosil-200 SiO_2_, 99.9%), *tetra*-butyl orthotitanate (TBOT, 98%), boric acid (H_3_BO_3_, 99.5%), deionized water and piperidine (PI, 99%) as the structure-directing agent. Typically, 17.8 g of PI was dissolved in 63 g of deionized water at room temperature and stirred for 30 min. 1.9 g of TBOT was slowly dripped into the solution, and the stirring was maintained for 1 h. Then, 11.6 g of H_3_BO_3_ was added while stirring. One hour later, 8.4 g of SiO_2_ was added into the solution, and vigorous stirring was maintained for another 2 h to form a homogeneous gel, with a molar composition of 1.0 SiO_2_ : 0.038 TiO_2_ : 0.67 B_2_O_3_ : 1.48 PI : 25.0 H_2_O. The gel was transferred into a Teflon-lined autoclave, which was clipped, rotated and heated at 403 K for 12 h and 443 K for 3 or 4 days (Fig. S1†) using special apparatus (Fig. S2†). After completion of the rota-crystallization, the autoclave was cooled down to ambient temperature. The solid product was recovered by filtration and washed with deionized water until pH ≈ 7, followed by drying at 373 K for 12 h (named as the Ti-HSZ precursor). The synthesis route is illustrated in Scheme 1. The same synthesis procedure was adopted for the synthesis of different samples under various conditions. For comparison, using conventional direct synthesis (stirring crystallization mode), the Ti-MWW zeolite was synthesized with a molar composition of 1.0 SiO_2_ : 0.038 TiO_2_ : 0.67 B_2_O_3_ : 1.40 PI : 19.0 H_2_O.^12^ The BET surface area of the calcined Ti-MWW zeolite was detected to be 536 m^2^ g^–1^. It is noteworthy that, to suit the present synthesis, the molar composition of the gel and the addition sequence of the raw materials were obviously varied from those for the synthesis of the traditional Ti-MWW zeolite.

An appropriate amount of the synthesized Ti-HSZ precursor was added into 50 ml of 2 M HNO_3_ solution and treated at 353 K for 20 h while stirring. Then, the solid product was recovered by filtration, washed with deionized water until pH ≈ 7, followed by drying at 373 K for 12 h (named as the Ti-HSZ catalyst).

### 2.3 Characterization of the materials

Powder XRD patterns were recorded on a Bruker D8A25 diffractometer with Cu Kα radiation (*λ* = 1.54184 Å) operating at 30 kV and 25 mA. UV-vis spectra were collected on a Shimadzu UV-visible UV-2550 spectrometer. FTIR spectra of the framework vibrations were recorded on a Perkin Elmer Spectrum One FTIR spectrophotometer. The morphology and size of the crystals were imaged on a JEOL JSM-6510A scanning electron microscope (SEM). TEM images were obtained using a FEI Tecnai G20 at an accelerating voltage of 200 kV. HRTEM images were obtained using a FEI Titan G^2^ 60-300 at an accelerating voltage of 200 kV. N_2_ adsorption/desorption isotherms were determined by a QuantachromeiQ-MP gas adsorption analyzer at 77.35 K. Before the nitrogen adsorption, the samples were degassed at 523 K for 3 h. The BET surface area was calculated based on the Brunauer–Emmett–Teller (BET) equation, and the pore distribution and total pore volume (at a relative pressure of *P*/*P*_0_ = 0.98) were calculated by the Barrett–Joyner–Halenda (BJH) method. Inductively coupled plasma (ICP) analysis was performed on a Perkin-Elmer Optima 3300Dv spectrometer. Elemental analyses (C, H, and N) were conducted on a Vario Micro elemental analyzer. Thermogravimetric analysis (TGA) was performed on a TASDTQ600 analyzer with a temperature-programmed rate of 10 K min^–1^ in air.

### 2.4 Catalytic testing

The epoxidation of allyl chloride with H_2_O_2_ was carried out in a 25 ml two-necked round-bottom-flask equipped with a reflux condenser under vigorous stirring and at atmospheric pressure. In a typical run, 5 ml of acetone, 30 mmol of allyl chloride, 30 mmol of H_2_O_2_ (30 wt%) and 200 mg of catalyst were mixed in the flask, and the reaction was run under magnetic stirring at 333 K. After completion of the reaction, the liquid products were separated by centrifugation and analyzed with a gas chromatograph (Shimadzu 2010), equipped with a 30 m capillary column (Rtx®-1) and an FID detector. The conversion of alkene and the selectivity of epoxide were calculated accordingly. In unspecified cases, the used catalyst (1.0 g) was washed with 60 ml of H_2_O_2_ solution (30 g 30% H_2_O_2_ in 30 g ethanol) at 343 K for 12 h. The solid catalyst was recovered by filtration and dried at 353 K for 6 h for the next use.

## 3. Results and discussion

**Fig. 1:**
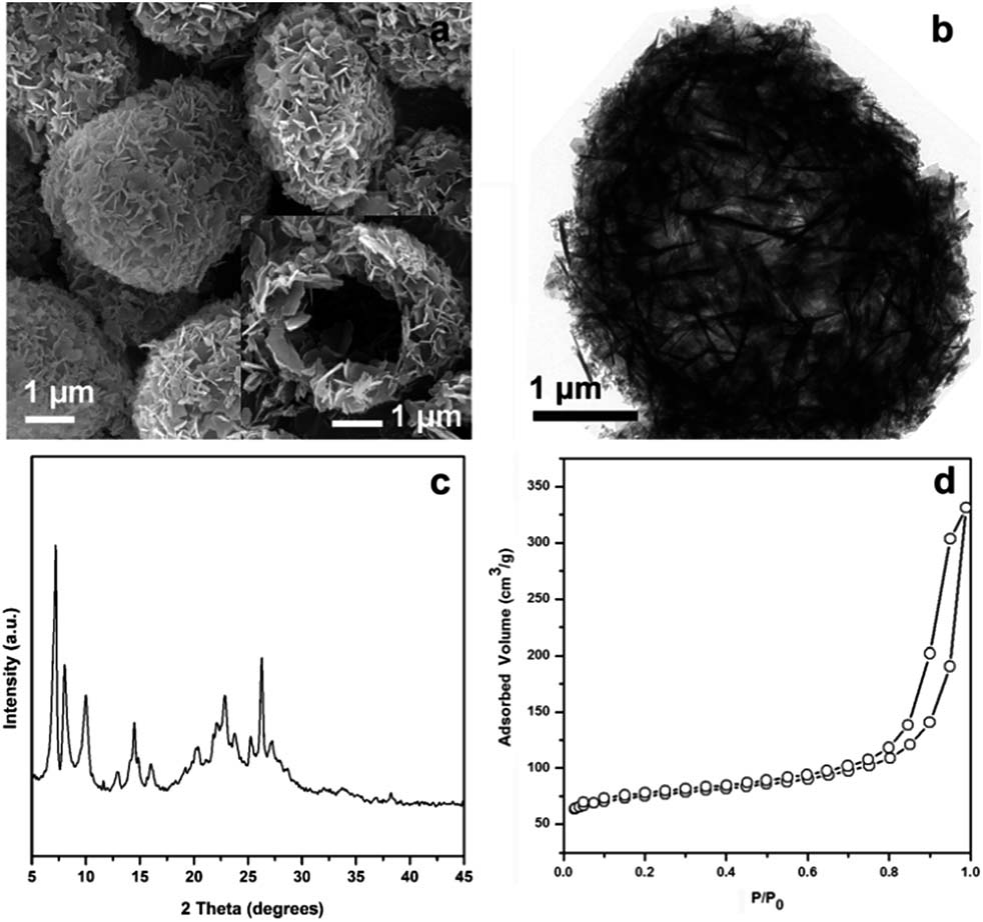
(a) SEM image, (b) TEM image, (c) XRD pattern, and (d) N_2_ adsorption–desorption isotherm of the Ti-HSZ catalyst synthesized at a rotating rate of 84 rpm.

The Ti-HSZ catalyst displays a hollownest morphology with a size of *ca.* 3 × 5 μm (Fig. 1a). Clearly, a microcavity is observed, with the shell constructed by randomly intergrown flaky crystals, as confirmed by TEM images (Fig. 1b). The XRD pattern of the Ti-HSZ catalyst (Fig. 1c) is typical of a MWW structure. The N_2_ adsorption–desorption isotherm of the material (Fig. 1d) shows a clear hysteresis loop at the relative pressure of *P*/*P*_0_ = 0.5–0.98, which is caused by capillary condensation in the textural stacking-pores assigned to intercrystalline voids in the shell of the crystalline hollownests.

**Fig. 2:**
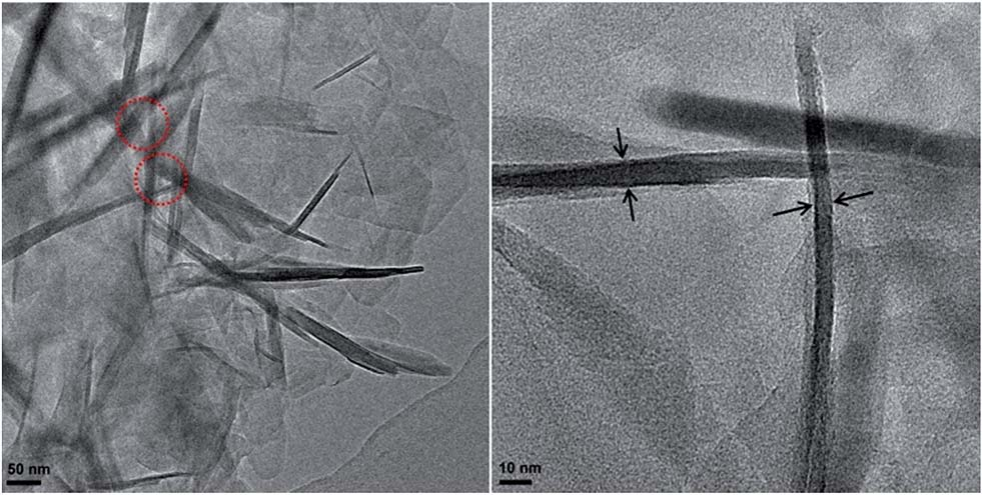
HRTEM images of the Ti-HSZ catalyst synthesized at a rotating rate of 84 rpm.

From the HRTEM images of the Ti-HSZ catalyst (Fig. 2), stacking-pores assigned to intercrystalline voids in the shell of the hollownests are observed, consistent with the N_2_ adsorption–desorption analysis results. The thickness of the MWW flaky crystals is less than 40 nm, typical of nanosheet crystals. The hollownest structure looks like a “house”, which is built from a large number of flaky crystals (as “bricks”) and stacking-pores (as “windows”). Such a spherical hollownest is entirely different from the flake morphology of the Ti-MWW zeolite synthesized by the conventional stirring crystallization method (Fig. S3†).^13^

The effect of stirring on the synthesis of mesoporous materials has been fully investigated.^22^–^24^ Stirring has been found to play an important role in inducing the formation of mesoporous materials with various morphologies, such as hollow spheres, nanoribbons, rods, hexagonal columns, and so on. For the synthesis of microporous zeolites, different crystallization conditions (static and stirring) show notable influences on the crystal morphologies and textural properties of the zeolites.^25^,^26^ Different from the direct synthesis and post structural rearrangement^12^–^16^ of the conventional Ti-MWW zeolite, the preparation of the Ti-HSZ precursor can be performed by a one-pot rota-crystallization in only 3.5 days, suggesting the efficiency of the rotating crystallization method. The XRD patterns of the Ti-HSZ precursors synthesized at different rotating rates are shown in Fig. S4a.† Distinctly, the sample prepared at a fast rotating rate (≥56 rpm) has a high crystallinity. In the IR spectra (Fig. S5†), the band at 960 cm^–1^, ascribable to Si–O–Ti stretching vibrations, is visible for the materials synthesized at fast rotating rates. The UV-vis spectra of the Ti-HSZ materials show one absorption band at 220 nm (Fig. S6†), which is assigned to the characteristic charge transfer from O^2–^ to the Ti^4+^ species incorporated in the zeolite framework.^13^

**Fig. 3:**
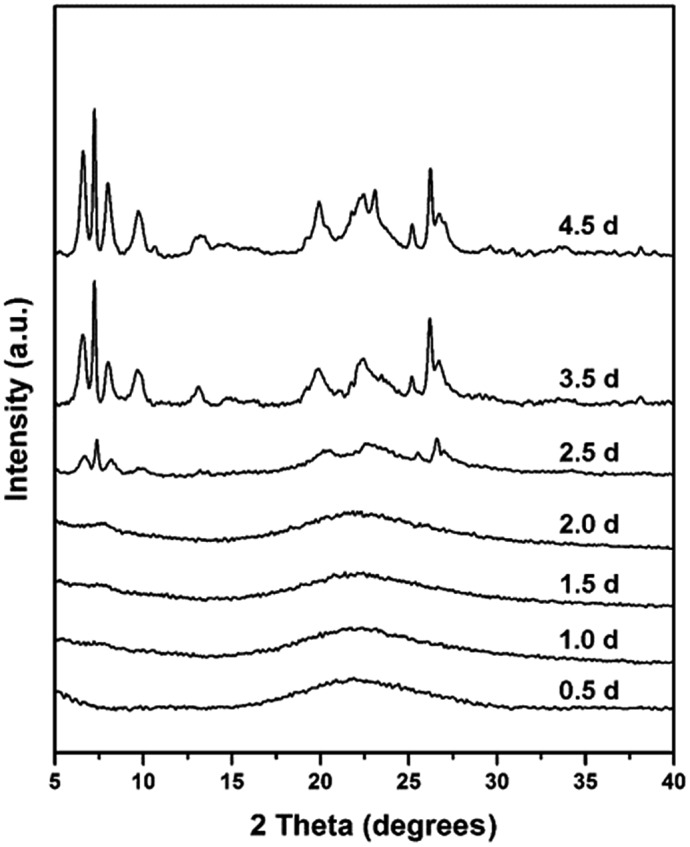
XRD patterns of the Ti-HSZ precursor synthesized at a rotating rate of 56 rpm and collected at different crystallization times.

**Fig. 4:**
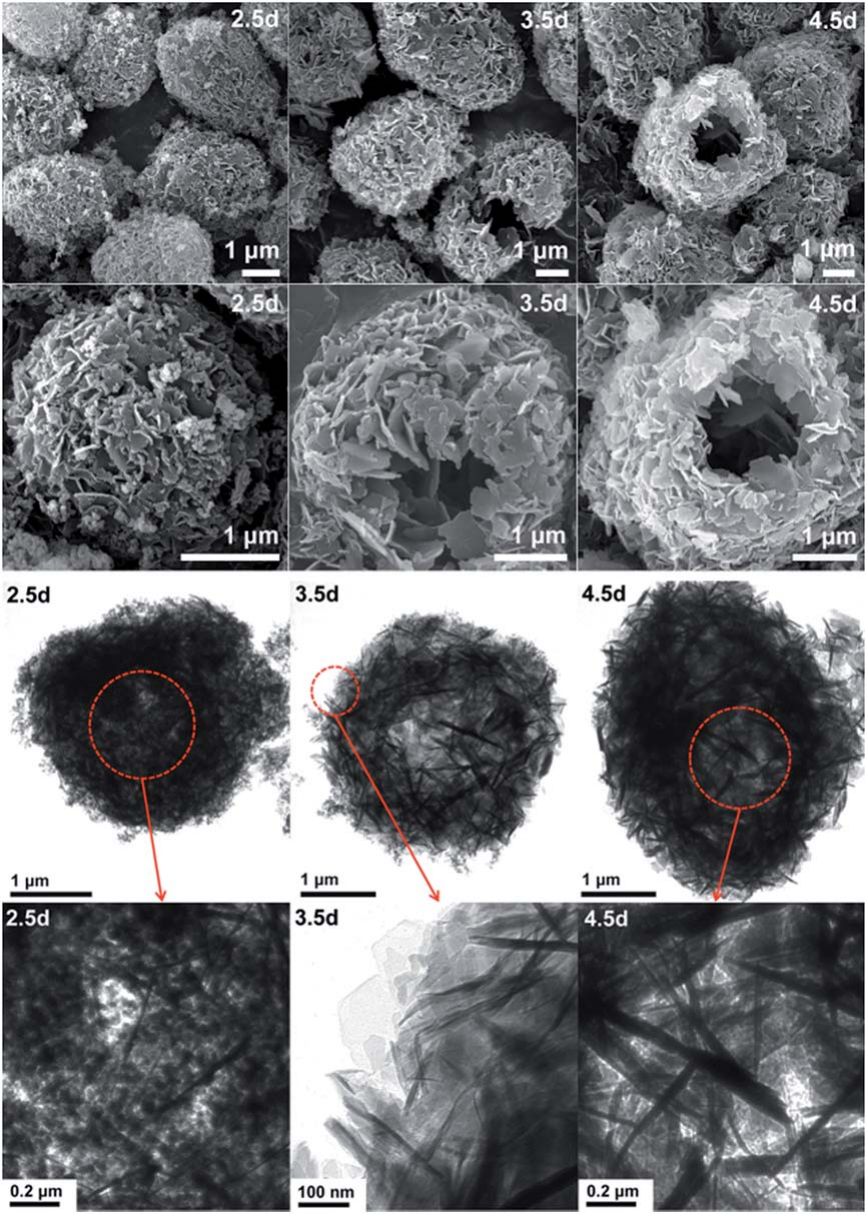
SEM (top) and TEM (bottom) images of the Ti-HSZ precursor synthesized at a rotating rate of 56 rpm and collected at different crystallization times.

In order to explore the crystallization process of the hollownest-structured zeolite, the effect of time on the crystallization of the Ti-HSZ precursor was investigated. Based on the XRD patterns of the Ti-HSZ precursors collected at different crystallization times (Fig. 3), the crystals began to form at 2.5 days. From the SEM and TEM images shown in Fig. 4, microsized aggregation spheres composed of disordered flaky crystals and a large amount of non-crystallized nano-SiO_2_ species have formed in 2.5 days, where the hollow centre can be observed. With an increase in the crystallization time to 3.5 days, a hollownest material with the shell composed of well-crystallized disordered intergrown flaky crystals is formed. The shell of the Ti-HSZ material crystallized after 4.5 days is more compact and thicker than that after 3.5 days, with a particle size of *ca.* 3.8 × 4.5 μm. The sample prepared at 56 rpm also presents a hollownest structure with a uniform size (Fig. 4), however, for that prepared at a relatively low rotating speed (28 rpm), irregular particles are observed (Fig. S4b†). This is probably because a fast rotating rate is beneficial for sufficient mixing of the various raw materials and the subsequent formation of microsized aggregation spheres of colloidal phase with a uniform size, which is rather important for the crystallization of the hollownest-structured zeolite.

**Scheme 2:**
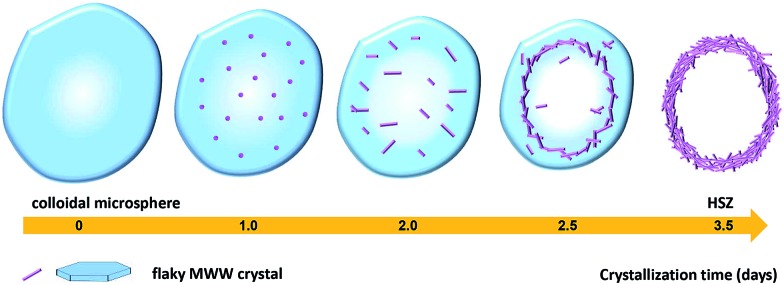
A possible formation mechanism of the Ti-HSZ precursor.

Based on the above results, a possible formation mechanism for the HSZ material is suggested (Scheme 2). Under fast rotating conditions (≥56 rpm), self-assembled microsized aggregation spheres of colloidal phase with a uniform size are formed due to the charge interaction and the moulding effect of rotation.^2^ In the initial 0–2 days, crystalline nuclei begin to form within the colloidal spheres. At 2.5 days, the crystallization process has occurred, resulting in the emergence of flaky crystals through the growth of various dissolved nutrients (Si, Ti, B, and PI source) surrounding the tiny nuclei. Such a repetition of the dissolution–growth process with crystallization time will lead to a number of MWW flaky crystals and the disordered intergrowth of these flaky crystals in the limited region of the original colloidal spheres. This produces a hollownest-structured zeolite with a number of intercrystalline voids in the shell of the microcavity. A too slow rotating rate is not beneficial to the occurrence of the above-mentioned process. More detailed investigations on the crystallization mechanism are ongoing, and the results will be published elsewhere.

The physical parameters of the Ti-HSZ catalysts are listed in Table 1. Compared with the Ti-MWW zeolite synthesized by the conventional method,^13^ the Ti-HSZ catalysts possess a large external surface area of 196–226 m^2^ g^–1^ and a small microporous surface area of 56–116 m^2^ g^–1^, in which the external/microporous ratio reaches 1.6–4.0 (Table 1). The BJH pore distribution indicates two average pore sizes of 3.6–3.8 and 17.1–17.4 nm based on the desorption branch of the isotherms.

**Table 1:** Physical parameters of the Ti-HSZ catalysts^*a*^

Rotating rate (rpm)/crystallization time (days)	Si/Ti ratio	Si/B ratio	BET surface area (m^2^ g^–1^)	Pore volume (cm^3^ g^–1^)	Exter. BET surface area (m^2^ g^–1^)	Micro. BET surface area (m^2^ g^–1^)
56/3.5	35	41	282	0.51	226	56
84/3.5	41	49	285	0.48	196	89
56/4.5	31	45	305	0.50	189	116

^*a*^The Si/B and Si/Ti ratios were analyzed by ICP, and the BET surface areas and pore volumes were calculated based on N_2_ adsorption analysis.

**Fig. 5:**
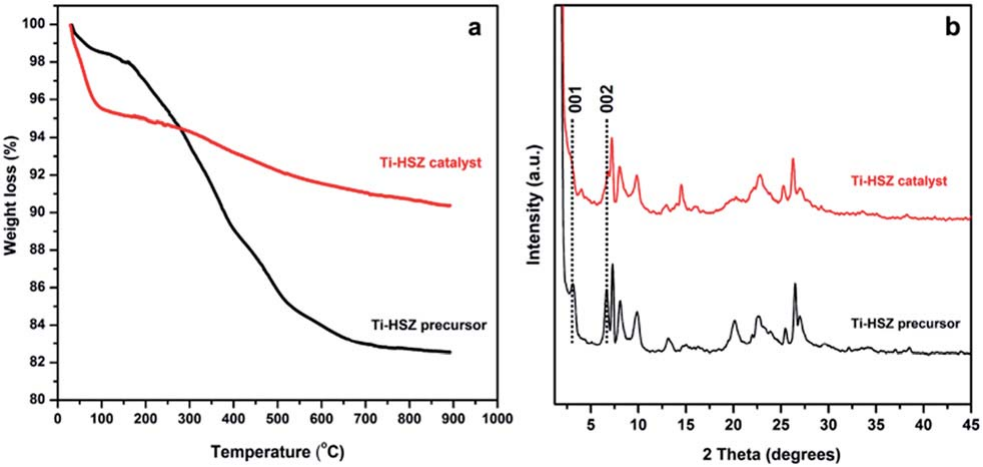
XRD patterns and TGA curves of the Ti-HSZ precursor and Ti-HSZ catalyst synthesized at a rotating rate of 56 rpm.

The TGA behaviours of the Ti-HSZ precursor and Ti-HSZ catalyst are shown in Fig. 5a. It was found that the total weight loss from room temperature to 1165 K was about 17.5 wt% for the Ti-HSZ precursor and 9.6 wt% for the Ti-HSZ catalyst. The weight loss below 473 K is due to the loss of physically and chemically adsorbed water, which is about 3.1 wt% for the Ti-HSZ precursor, and 5.1 wt% for the Ti-HSZ catalyst. The lower weight loss for the Ti-HSZ precursor is due to a higher hydrophobicity caused by the existence of more organic PI molecules. The weight loss in the range of 473–773 K is due to the combustion of organic PI molecules, which is about 10.8 and 1.5 wt% for the Ti-HSZ precursor and catalyst, respectively, which are similar values to the organic content of 10.7 and 1.3 wt% obtained from the element analysis (Table S1†), respectively. The weight loss at >773 K is coming from the thermal condensation of Si–OH groups. From the XRD patterns shown in Fig. 5b, the intensity of the 001 and 002 diffraction lines (characteristic of a layered structure for the Ti-HSZ precursor) is decreased as a result of the partial disappearance of the lamellar structure for the Ti-HSZ catalyst (acid-washed and dried precursor), implying the partial removal of the structure directing agent PI intercalated in the sheets and between the layers, consistent with the TGA analysis results.

**Scheme 3:**
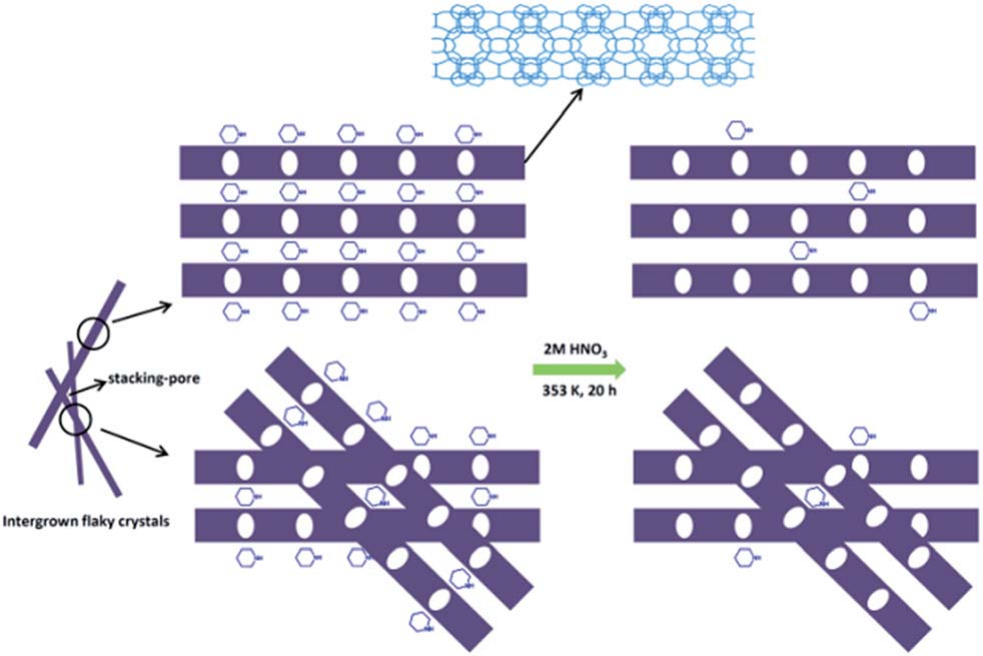
Acid treatment of the Ti-HSZ catalyst.

Furthermore, the elemental analysis was also conducted and the results are listed in Table S1.† About 90.4% of PI occluded in the Ti-HSZ precursor can be removed by a simple acid-washing process, implying the efficiency of acid treatment for the removal of the structure-directing agents from the hollownest-structured zeolite, as proven by the TGA analysis. Scheme 3 demonstrates the acid treatment process. Due to the large external surface area and the existence of a large number of stacking-pores in the shell, the majority of PI molecules located in the Ti-HSZ nanosheet crystals can be removed by acid-washing.

In the epoxidation of allyl chloride with hydrogen peroxide to epichlorohydrin, the Ti-HSZ catalyst presents a high allyl chloride conversion of 96.7 mol% and an epichlorohydrin selectivity of 100% (Table 2), implying the efficiency of the catalyst and the acid treatment.

**Table 2:** Epoxidation of allyl chloride with hydrogen peroxide to epichlorohydrin over the Ti-HSZ catalyst^*a*^

No.	Rotating rate (rpm)	Crystallization time (days)	Conversion (mol%)	Selectivity (%)
1	56	3.5	95.8	100
2	84	3.5	96.7	100
3	56	4.5	94.4	100
4^*b*^	—	7	94.8	100
5^*c*^	—	7	65.6	100

^*a*^Reaction conditions: allyl chloride (30 mmol), H_2_O_2_ (30 mmol), catalyst (200 mg), acetone (5.0 ml), reaction temperature 333 K, reaction time 8 h.

^*b*^Traditional Ti-MWW zeolite catalyst calcined at 773 K in air for 4 hours.

^*c*^Only acid-washed and dried Ti-MWW catalyst.

**Fig. 6:**
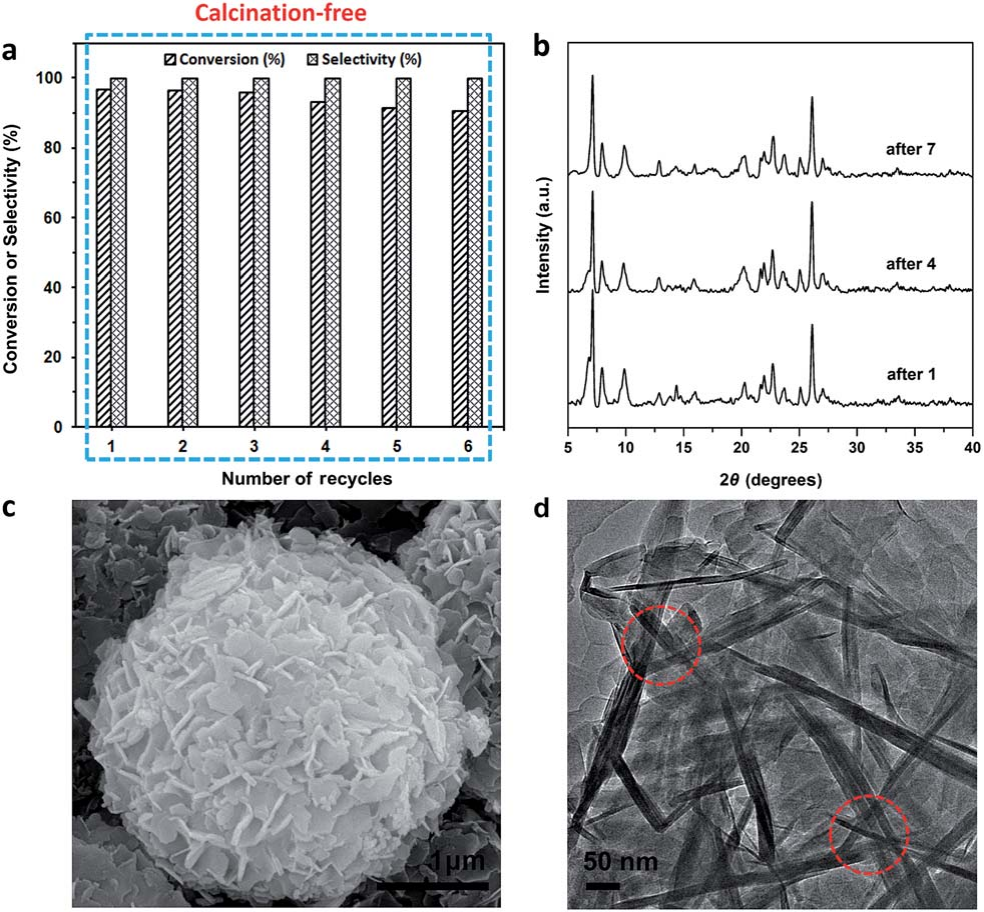
(a) Recycling tests with the Ti-HSZ catalyst (reaction conditions: allyl chloride (30 mmol), H_2_O_2_ (30 mmol), catalyst (200 mg), acetone (5 ml), reaction temperature 333 K, reaction time 8 h). (b) XRD patterns of the catalysts reused 1, 4, and 7 times. (c) SEM image of the catalyst reused 6 times. (d) HRTEM image of the Ti-HSZ catalyst washed with H_2_O_2_/ethanol solution.

The recycling experiments were conducted with the Ti-HSZ catalyst synthesized at a rotating rate of 56 rpm for 3.5 days. In our work, the used catalyst (1.0 g) was washed with 60 ml of H_2_O_2_/ethanol solution (30 g 30% H_2_O_2_ in 30 g ethanol) at 343 K for 12 h. The solid catalyst was recovered by filtration and dried at 353 K for 6 h before the next use. As observed from Fig. 6a, the reuse of this Ti-HSZ catalyst (6 times) did not appreciably decrease the conversion of allyl chloride and the selectivity of epichlorohydrin – all the reactions maintained a small fluctuation in the conversion of about 90.8–96.7 mol% and an almost constant selectivity of 100%, suggesting the excellent heterogeneous recyclability of the calcination-free Ti-HSZ catalyst.

In order to make a clear comparison, the traditional Ti-MWW catalyst was prepared from the precursor by pre-treatment with 2 M HNO_3_, drying and subsequent calcination at 773 K or by only acid washing and drying. As listed in Table 2, under identical conditions, the conversion of allyl chloride over the pre-calcined Ti-MWW catalyst reached 94.8 mol% with 100% epoxide selectivity, comparable to that over the Ti-HSZ catalysts in this work. Additionally, the Ti-MWW catalyst shows an excellent recyclable stability when it is activated by calcination in every recycle (Fig. S7†). However, this pre-calcined Ti-MWW catalyst cannot maintain a nice stability during recycling with regeneration using only the H_2_O_2_/ethanol solution treatment applied in this work. One can see from Table S2† that the conversion of allyl chloride over Ti-MWW dropped to 39.7 mol% in the 4^th^ cycle. This is mainly assigned to the difficulty in washing off the organic molecules trapped in the microporous channels of the Ti-MWW catalyst, from which 10.7 wt% organic C is detected by elemental analysis. When only acid-washed and dried Ti-MWW is used as the catalyst, the initial conversion of allyl chloride is largely decreased to 65.6 mol% (Table 2). As reported in the literature,^13^ over a conventional Ti-MWW catalyst, the conversion of allyl chloride was dramatically decreased with the reaction–regeneration cycles when the used catalyst was merely washed with acetone and then dried. Only when the used catalyst was activated by calcination in air at 773 K from the very beginning does the conversion of allyl chloride decrease slowly, suggesting that the deactivation of the acetone-washed catalyst could be due to the deposition of heavy organic compounds with high boiling points inside the channels.

The XRD patterns (Fig. 6b), the SEM images (Fig. 6c) of the used catalysts, and the HRTEM images (Fig. 6d) of the Ti-HSZ catalyst washed with H_2_O_2_/ethanol solution show the stability of the hollownest-structured zeolite catalyst. The excellent heterogeneous recyclability of the Ti-HSZ catalyst is ascribed to the stability of the structure with the number of stacking-pores in the shell beneficial for the removal of organic compounds trapped in the catalyst.

**Fig. 7:**
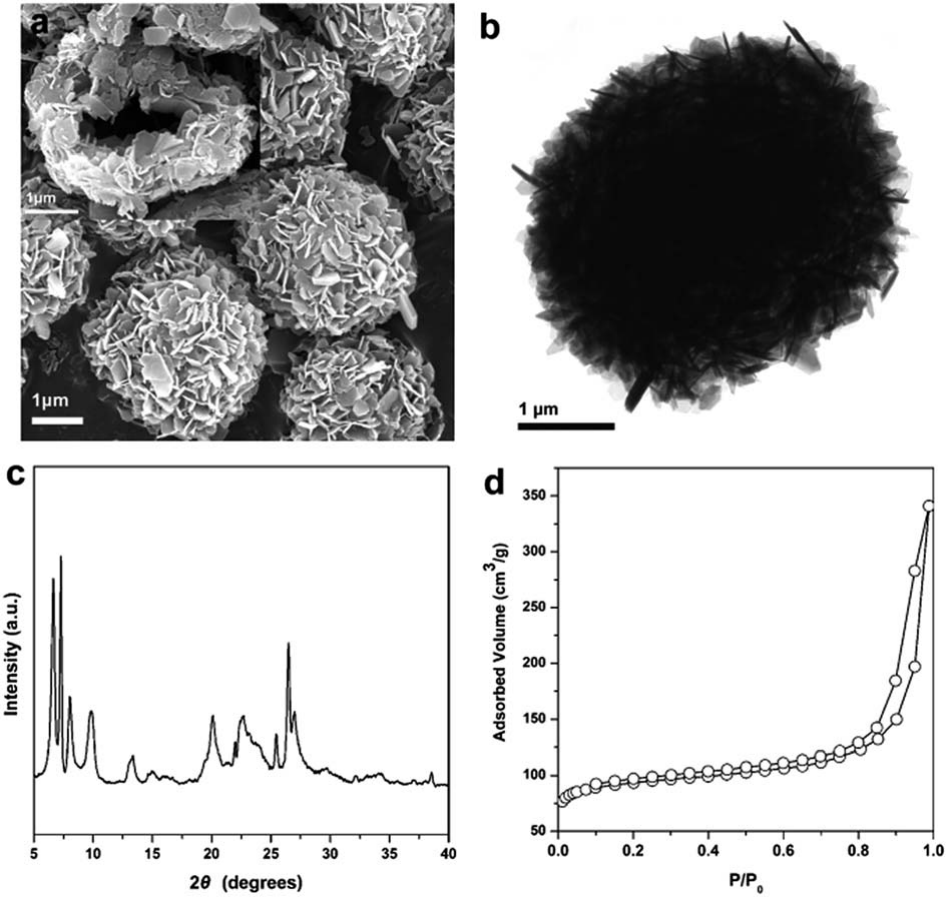
Characterization of the B-HSZ sample: (a) SEM image, (b) TEM image, (c) XRD pattern, and (d) N_2_ adsorption–desorption isotherm.

By using the same synthesis method, a B-containing hollownest-structured zeolite (B-HSZ) was successfully prepared, as revealed by various characterization methods, including the SEM image, TEM image, XRD pattern and N_2_ adsorption–desorption isotherm in Fig. 7. This synthesized B-HSZ material possesses a large external surface area of 199 m^2^ g^–1^, a small microporous surface area of 94 m^2^ g^–1^, and a large pore volume of 0.52 cm^3^ g^–1^. In our work, micro–meso–macroporous HSZ samples with a large pore volume of 0.48–0.52 cm^3^ g^–1^ are crystallized without any core template. These values are much larger than the pore volume of 0.15–0.16 cm^3^ g^–1^ of hierarchical MCM-22 microspheres prepared by using carbon black microspheres as a hard core template.^20^

## 4. Conclusions

A micro–meso–macroporous hierarchical Ti-containing hollownest-structured zeolite precursor, with the shell composed of autogenously intergrown MWW nanosheet crystals, was first synthesized in only 3.5 days by a one-step hydrothermal rota-crystallization method without using any solid or supramolecules. Calcination at high temperatures is not necessary for the treatment of the precursor and for the reaction–regeneration recycling. Using only acid-washing, the majority of the piperidine molecules can be removed from the zeolite precursor, and the resultant Ti-HSZ catalyst presents an excellent catalytic activity for the epoxidation of alkenes with 30% H_2_O_2_. By washing with H_2_O_2_/ethanol solution, the reuse of this Ti-HSZ catalyst (6 times) does not appreciably decrease the conversion of the allyl chloride and the selectivity of the epichlorohydrin. The excellent catalytic activity and recyclable stability of the Ti-HSZ catalyst are attributable to the unique morphology of the material with a large external surface area and the existence of a number of stacking-pores in the shell, which are beneficial for the removal of PI and organic compounds in the zeolite and the superiority of the hollownest-structured zeolite.

## Supplementary Material

Supplementary information
