# Supplementary material for: One-pot rota-crystallized hollownest-structured Ti–zeolite: a calcination-free and recyclable catalytic material
Source: Chem Sci. 2016 May 4;7(8):4966–72. doi: 10.1039/c6sc01735e (PMC6018383; doi:10.1039/c6sc01735e)
Supplement: Supplementary file 1 [file SC-007-C6SC01735E-s001.pdf]

Electronic Supplementary Material (ESI) for Chemical Science.
This journal is © The Royal Society of Chemistry 2016

Supporting Information

Table S1 The elemental analysis results of Ti-HSZ precursor (synthesized at rotating rate of 56 rpm for 3.5 days) and Ti-HSZ catalyst.

Sample	N (wt%)	C (wt%)	H (wt%)
Ti-HSZ precursor	1.67	7.53	1.57
Ti-HSZ catalyst	0.16	0.89	0.30

Table S2 Reuse of traditional Ti-MWW zeolite catalyst in the epoxidation of ally chloride with hydrogen peroxide to epichlorohydrin under the present reaction and regeneration conditions.[a]

Reused times	Conv. (mol%)	Sele. (%)
Fresh	94.8	100
1	85.3	99.9
2	73.1	99.9
3	58.2	99.9
4	39.7	99.9

[a] Fresh catalyst is prepared by calcination at 773 K in air for 4 hours ;
Reaction conditions: allyl chloride, 30 mmol; H_2O_2, 30 mmol; catalyst, 200 mg; acetone, 5 ml, reaction temperature, 333 K; reaction time, 8 h;
Regeneration: the used catalyst (1 g) was washed by 60 ml of H_2O_2 solution (30 g 30%H_2O_2 in 30 g ethanol) at 343 K for 12 h. The solid catalyst was recovered by filtration and dried at 353 K for 6 h for next use.

Captions:

Figure S1 Three hydrothermal crystallization modes: (a) static, (b) stirring, and (c) rotating in this work.

Figure S2 Crystallization apparatus in this work.

Figure S3 Characterization of Ti-MWW sample synthesized by conventional stirring crystallization mode: (a) SEM image, and (b) XRD pattern.

Figure S4 XRD patterns of Ti-HSZ catalyst synthesized at different rotating rates for 3.5 days (a) and SEM images of Ti-HSZ catalyst synthesized at rotating rates of 28 rpm (b).

Figure S5 FTIR spectra of Ti-HSZ catalysts synthesized at different rotating rates.

Figure S6 UV-vis spectra of Ti-HSZ catalysts crystallized at different rotating rates.

Figure S7 Recycling tests over traditional Ti-MWW catalyst. (Reaction conditions: allyl chloride, 30 mmol; H_2O_2, 30 mmol; catalyst, 200 mg; acetone, 5 ml; reaction temperature, 333 K; reaction time, 8 h.)

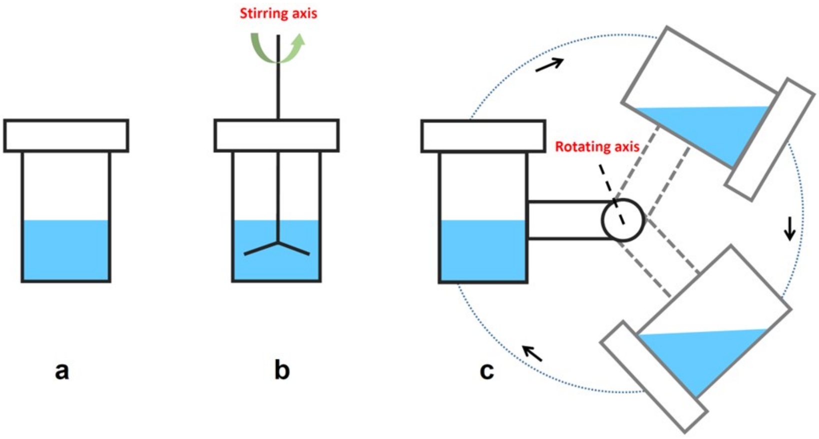

Figure S1

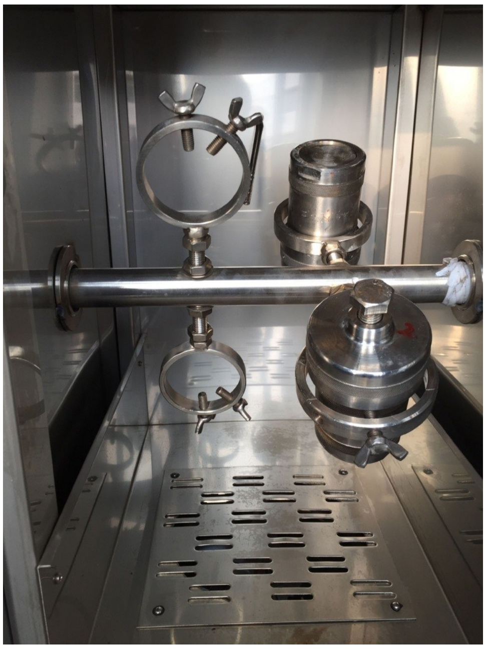

Figure S2

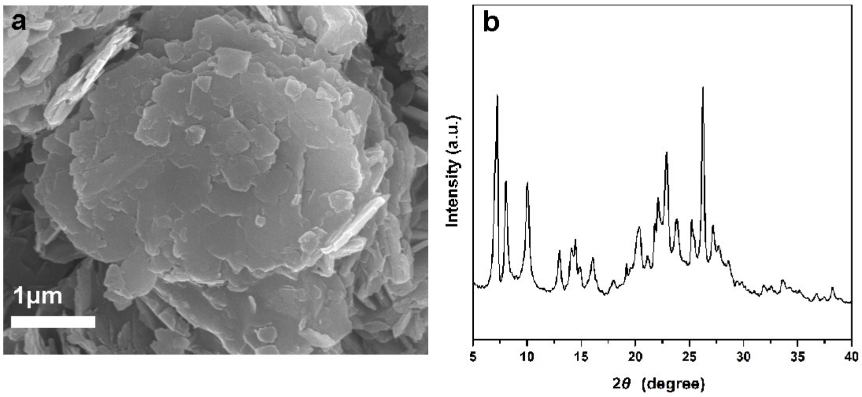

Figure S3

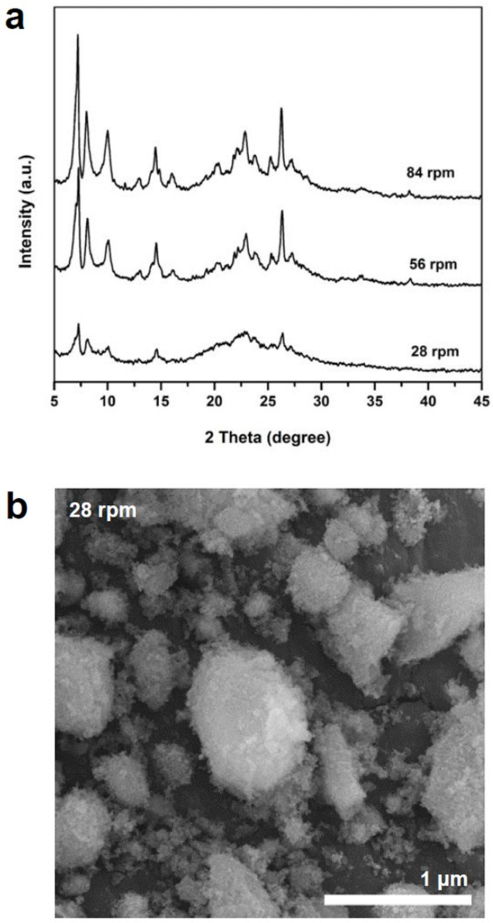
a
Intensity (a.u.)
84 rpm
56 rpm
28 rpm
5
10
15
20
25
30
35
40
45
2 Theta (degree)
b
28 rpm
1 μm

Figure S4

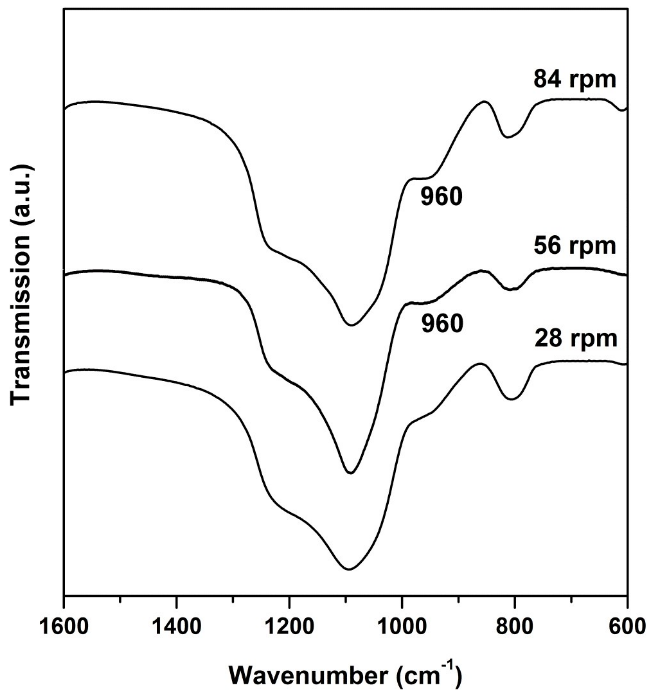
84 rpm
960
56 rpm
960
28 rpm
Transmission (a.u.)
1600
1400
1200
1000
800
600
Wavenumber (cm-1)

Figure S5

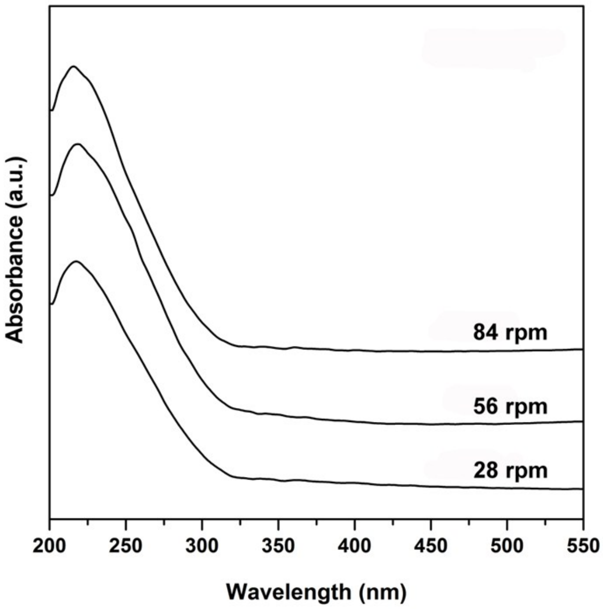
Absorbance (a.u.)
84 rpm
56 rpm
28 rpm
200
250
300
350
400
450
500
550
Wavelength (nm)

Figure S6

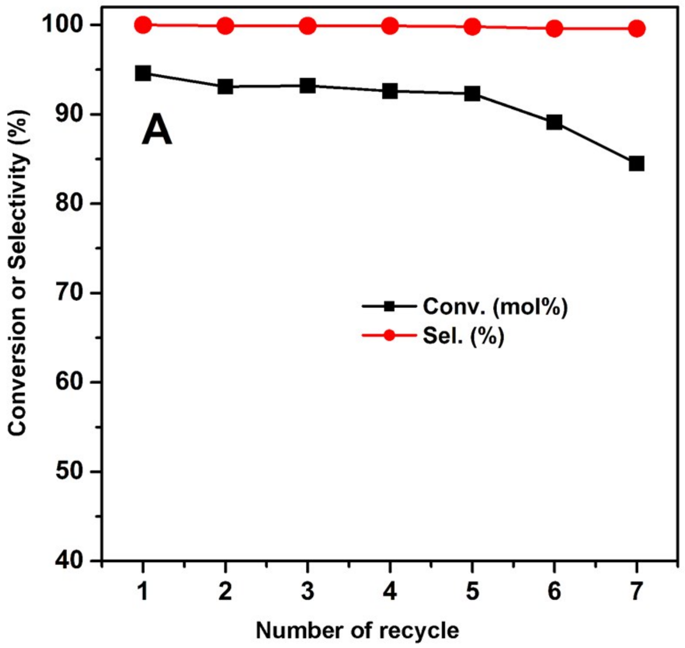
100
90
80
70
60
50
40
A
Conversion or Selectivity (%)
Conv. (mol%)
Sel. (%)
1
2
3
4
5
6
7
Number of recycle

Figure S7
